# Vitamin D levels in early and middle pregnancy and preeclampsia: a systematic review and meta-analysis

**DOI:** 10.3389/fmed.2025.1535359

**Published:** 2025-10-23

**Authors:** Faezeh Zakerinasab, Qumars Behfar, Hussam Daghistani, Arina Ansari, Reza Hossein Zadeh, Rasoul Hossein Zadeh, Amirhesam Amirbeik

**Affiliations:** ^1^Mashhad University of Medical Sciences, Mashhad, Iran; ^2^Department of Neurology, Faculty of Medicine and University Hospital Cologne, University of Cologne, Cologne, Germany; ^3^Department of Clinical Biochemistry, Faculty of Medicine, King Abdulaziz University, Jeddah, Saudi Arabia; ^4^Student Research Committee, School of Medicine, North Khorasan University of Medical Sciences, Bojnurd, Iran; ^5^Student Research Committee, School of Medicine, Mashhad University of Medical Sciences, Mashhad, Iran

**Keywords:** vitamin D, preeclampsia, pregnancy, systematic review, meta-analysis

## Abstract

**Background and aims:**

Previous studies have indicated a potential association between low vitamin D levels in early pregnancy and an increased risk of hypertensive disorders, including preeclampsia. Given the substantial maternal and fetal morbidity associated with preeclampsia, identifying preventive strategies is crucial. This meta-analysis aimed to evaluate the relationship between vitamin D status in early and middle pregnancy and the development of preeclampsia.

**Methods:**

A comprehensive literature search of PubMed, Scopus, Web of Science, and Cochrane Library databases was conducted up to June 23, 2023, to identify relevant observational studies. Included studies were assessed for methodological quality, and data on maternal vitamin D concentration and the risk of preeclampsia were extracted.

**Results:**

Twenty-nine observational studies with 74,061 participants were included. Women with preeclampsia had significantly lower vitamin D levels than those without (SMD −0.28, 95% CI −0.39 to −0.17, *p* < 0.001). Although lower vitamin D levels showed a trend toward higher preeclampsia risk, pooled odds ratios for insufficiency (OR 1.05, 95% CI 0.78–1.42) and deficiency (OR 1.25, 95% CI 0.89–1.76) were not statistically significant. Subgroup analyses suggested a possible dose–response relationship, especially when vitamin D was measured in early or mid-pregnancy. Additional analyses by assay method, cut-off definitions, region, and study design also supported an association between lower vitamin D and preeclampsia risk.

**Conclusion:**

The findings of this meta-analysis suggest a potential association between low maternal vitamin D levels and an increased risk of preeclampsia, particularly when measured prior to the late pregnancy. However, the precise timing of this association requires further investigation. To definitively establish the role of vitamin D supplementation in preventing preeclampsia, well-designed randomized controlled trials are needed to determine optimal dosage and timing of intervention, as well as to assess cost-effectiveness.

**Systematic review registration:**

Registered in OSF: https://osf.io/qwh6a (Unique ID: qwh6a).

## Background

Preeclampsia is a severe pregnancy complication characterized by new-onset high blood pressure and proteinuria, often accompanied by damage to other organs. This condition typically develops after 20 weeks of gestation and is a leading cause of maternal and fetal morbidity and mortality worldwide (1). While its prevalence varies globally, it significantly impacts approximately 4.6% of pregnancies, with higher rates reported in developing countries (2). The course of preeclampsia can be dreadful if neglected, leading to adverse outcomes such as placental abruption, preterm delivery, and HELLP syndrome for the mother and preterm birth, stillbirth, low birth weight, and small for gestational age for the fetus (3). The precise etiology of preeclampsia remains elusive. However, it is widely accepted that abnormal placental development plays a central role, triggering a cascade of events including oxidative stress and systemic inflammation (4, 5). Currently, the only definitive cure for preeclampsia is delivery. Pharmacological interventions are primarily aimed at managing symptoms and preventing complications until delivery can be safely achieved (6). Recent research has focused on the potential link between maternal vitamin D status and the development of preeclampsia. Vitamin D is known to play a critical role in regulating genes involved in placental function, implantation, blood vessel development, and immune response. During pregnancy, the placenta is a key site for vitamin D activation, expressing enzymes like CYP27B1 and CYP24A1, as well as the vitamin D receptor (VDR), indicating its role in local vitamin D homeostasis (7). Gene-based studies show that vitamin D supplementation or status is linked to changes in placental gene expression. These include increased activity in amino acid transporter genes (8), reduced levels of an antiangiogenic factor tied to preeclampsia (9), and better regulation of inflammatory responses during immune challenges (10).

Moreover, low vitamin D levels during early pregnancy have been linked to an increased risk of hypertensive disorders and can also influence cholesterol levels throughout gestation (11). Vitamin D deficiency is prevalent among pregnant women, with estimates suggesting that up to 40% of this population is affected (12). Given the multiple factors influencing vitamin D levels, including diet, fortification, skin pigmentation, sun exposure, and genetics, it is considered a potential modifiable risk factor for preeclampsia prevention (13). Despite the potential association between vitamin D status and preeclampsia, clear guidelines for vitamin D supplementation during pregnancy are lacking. Observational studies have yielded inconsistent results, likely due to factors such as small sample sizes, varying definitions of vitamin D deficiency, and the often late detection of low vitamin D levels. These challenges hinder the establishment of a definitive causal relationship between vitamin D and preeclampsia prevention. The most recent meta-analysis, conducted by Hu et al. (14) in 2021, examined the association between vitamin D levels and preeclampsia before the late stage of pregnancy. Previous meta-analyses have reported an inverse relationship between vitamin D status and preeclampsia risk (15–17). However, the evolving body of evidence necessitates an updated assessment. Given the inconsistencies in the literature and the emergence of new studies, we conducted a comprehensive systematic review and meta-analysis to examine the association between vitamin D status in early and middle pregnancy and the development of preeclampsia.

## Methods

Our methodology adheres to the PRISMA guidelines (Preferred Reporting Items for Systematic Reviews and Meta-analyses (18). The research protocol of this review was registered on the Open Science Framework.^1^

### Eligibility criteria, information sources, search strategy

An advanced literature search was performed up to June 23, 2023 to retrieve relevant articles from following databases: PubMed, Scopus, Web of Science, and Cochrane Library. A comprehensive search strategy was developed using keywords and Medical Subject Headings (MeSH) terms related to vitamin D, pregnancy, and preeclampsia. These terms were combined using the Boolean operator “AND” without imposing restrictions on publication date, type, or language. Database-specific search syntax was employed for each database. To enhance the search yield, reference lists of relevant systematic reviews were manually screened. Two independent reviewers conducted the search process, with discrepancies resolved through consensus.

Early pregnancy was classified as gestational age less than 14 weeks, middle pregnancy as gestational age between 14 and 27 weeks, and late pregnancy as gestational age 28 weeks or greater (19). For studies to be considered in this meta-analysis, the following criteria should be met:

1. Observational methodology (in order to exclude the confounding effect of any intervention).
2. The main interest was to assess the link between vitamin D levels and risk of preeclampsia.
3. Study population consisted of pregnant women before the 28 weeks of gestational age.
4. Preeclampsia was defined by the new-onset of gestational hypertension and proteinuria.
5. Threshold levels for vitamin D sufficiency and insufficiency were outlined.

Studies employing methodologies other than observational designs, those conducted on animal models, or those involving pregnant women with pre-existing conditions or outcomes other than preeclampsia were excluded from the analysis.

### Study selection, data extraction and study quality assessment

Two independent reviewers screened titles and abstracts of all retrieved studies to assess eligibility for the meta-analysis. Studies not meeting the inclusion criteria were excluded. Subsequently, full texts of potentially eligible studies were reviewed for final selection. Next, the following items were obtained for extraction in four sets: (1) Study characteristics (i.e., authors, location, year, and type of study), (2) patient-specific factors (i.e., gestational age), (3) Study Design (i.e., number of participants, method and period of sampling, confounding factors), (4). Outcomes (i.e., risk of preeclampsia and vitamin D concentration). Serum vitamin D concentrations reported in ng/mL were converted to nmol/L using the standard conversion factor (1 ng/mL = 2.5 nmol/L). *Critical appraisal* of included studies was performed by two reviewers using the Joanna Briggs Institute (JBI) checklists designed specifically for cohort, case–control, and analytical cross-sectional studies.^2^ A third reviewer was involved to resolve any discrepancies in selection or data extraction.

### Data synthesis

We used STATA 13.1 software, developed by StataCorp LP in College Station, TX, USA, for our data analysis. Results were reported as pooled odds ratios (ORs) with a 95% confidence interval, visualized in a forest plot. We evaluated heterogeneity among the eligible studies using the *I*^2^ statistic (20). For the primary meta-analyses and meta-regression models, between-study variance (τ^2^) was estimated using the restricted maximum likelihood (REML) method, the DerSimonian–Laird random-effects model was additionally applied as a sensitivity estimator when substantial heterogeneity was present (*I*^2^ > 50%) (21). To explore potential sources of heterogeneity, we performed subgroup and meta-regression analyses across parameters including assay method, vitamin D threshold, geographic region, and study design. Furthermore, we conducted a sensitivity analysis, excluding one study at a time and repeating the meta-analysis. This enabled us to ensure the stability of our findings. Finally, to investigate the potential for publication bias, we adopted visual inspection of funnel plot symmetry and Egger’s regression analysis (22).

## Results

### Study selection

**Figure 1:**
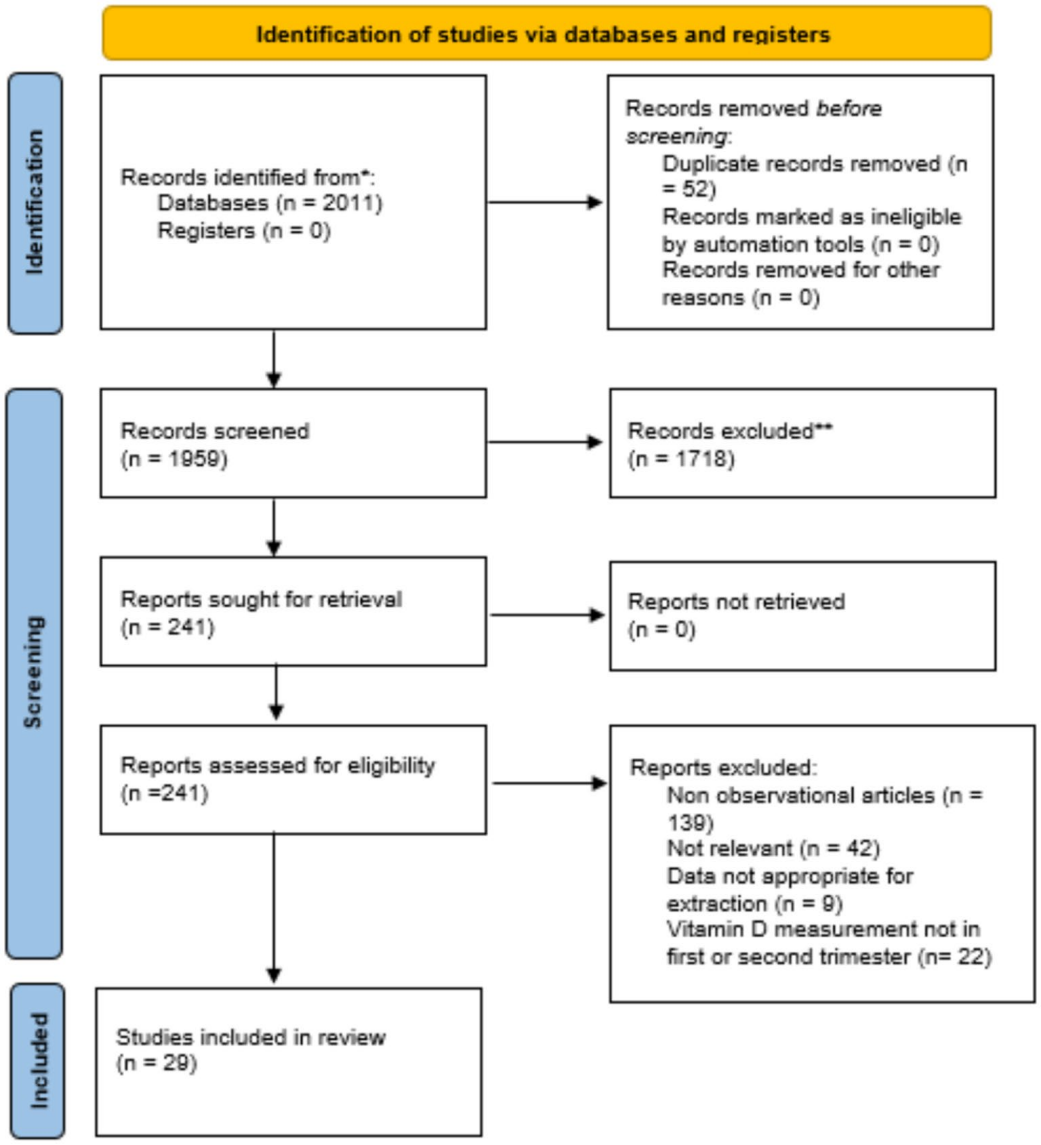
PRISMA flow diagram for current systematic.

An initial search as described in Table 1 yielded 2,011 studies, of which 52 duplicates were removed, leaving 1,959 records. Abstract screening excluded 1,718 studies, resulting in 241 articles for full-text review. After this stage, 212 studies were eliminated, leaving 29 eligible for meta-analysis (Figure 1). These studies, published between 2007 and 2023, included a total of 74,061 pregnant women with vitamin D measurements obtained during the early or middle pregnancy.

**Table 1:** Search strategy for selected databases.

Database	Search strategy	Result
Pubmed	#1: ((“vitamin D”[MeSH Terms]) OR (“vitamin D”[Title/Abstract]) OR (“cholecalciferol”[Title/Abstract]) OR (“ergocalciferol”[Title/Abstract])) #2: (“pregnancy”[MeSH Terms]) OR (“pregnancy”[Title/Abstract]) #3: (“pre-eclampsia”[MeSH Terms]) OR (“eclampsia”[MeSH Terms]) OR (“Hypertension, Pregnancy-Induced”[MeSH Terms]) OR (“preeclampsia”[Title/Abstract]) OR (“hypertensive disorder of pregnancy”[Title/Abstract]) OR (“gestational hypertension”[Title/Abstract]) OR (“gestational hypertensive disorder”[Title/Abstract]) OR (“hypertensive disorder during pregnancy”[Title/Abstract]) OR (“pregnancy induced hypertension”[Title/Abstract]) OR (“pre-eclamptic toxaemia”[Title/Abstract]) Final search: #1 AND #2 AND #3	496
Cochrane library	#1: MeSH descriptor: [Vitamin D] explode all trees OR (vitamin D):ti,ab,kw OR (cholecalciferol):ti,ab,kw OR (ergocalciferol):ti,ab,kw #2: MeSH descriptor: [Pregnancy] explode all trees OR (pregnancy):ti,ab,kw #3: MeSH descriptor: [Pre-Eclampsia] explode all trees OR MeSH descriptor: [Eclampsia] explode all trees OR MeSH descriptor: [Hypertension, Pregnancy-Induced] explode all trees OR (preeclampsia):ti,ab,kw OR (hypertensive disorder of pregnancy):ti,ab,kw OR (gestational hypertension):ti,ab,kw OR (gestational hypertensive disorder):ti,ab,kw OR (hypertensive disorder during pregnancy):ti,ab,kw OR (pregnancy induced hypertension):ti,ab,kw OR (pre-eclamptic toxaemia):ti,ab,kw Final search: #1 AND #2 AND #3	124
Scopus	#1: TITLE-ABS-KEY (“vitamin D”) OR TITLE-ABS-KEY (cholecalciferol) OR TITLE-ABS-KEY (ergocalciferol) #2: TITLE-ABS-KEY (“pregnant” OR “pregnancy”) #3: TITLE-ABS-KEY (“pre-eclampsia” OR eclampsia OR preeclampsia OR “gestational hypertension” OR “gestational hypertensive disorder” OR “hypertensive disorder during pregnancy” OR “pregnancy induced hypertension” OR pih OR “pre-eclamptic toxaemia”) Final search: #1 AND #2 AND #3	903
Web of sciences	#1: TS = (“vitamin D”) OR TS = (cholecalciferol) OR TS = (ergocalciferol) #2: TS = (pregnancy) #3: TS = (“pre-eclampsia” OR eclampsia OR preeclampsia OR “gestational hypertension” OR “gestational hypertensive disorder” OR “hypertensive disorder during pregnancy” OR “pregnancy induced hypertension” OR pih OR “pre-eclamptic toxaemia”) Final search: #1 AND #2 AND #3	479

Most studies used cutoff values of 75 nmol/L and 50 nmol/L to define vitamin D sufficiency and insufficiency, respectively, in accordance with Endocrine Society recommendations (23). However, four studies defined vitamin D deficiency as levels below 37.5 nmol/L based on national or local guidelines (24–27). Due to the bias of cofounders, most of the studies included in this analysis have reported data in an adjusted format. However, there is notable heterogeneity regarding the number and types of controlled variables.

### Study characteristics

Thirteen studies quantified maternal serum concentrations of vitamin D at early pregnancy (25, 26, 28–38), six studies at middle pregnancy (24, 39–43), and 10 other studies measured maternal vitamin D concentrations during a time period overlapping between the two (<22 weeks) (27, 44–52). Out of articles included, eight were carried out in USA (24, 25, 27, 32, 39, 41, 48, 49), five were from China (35, 38, 42, 43, 52), four from Canada (28, 40, 44, 50), two from Australia and New Zealand (26, 45), one from India (36), and the rest were in Europe (29–31, 33, 34, 37, 46, 47, 51). A summary of the included studies is presented in Table 2, with detailed characteristics available in Supplementary Table S1.

**Table 2:** Summary of included studies on maternal vitamin D levels and preeclampsia risk.

Author (year)	Country	Study type	Method	Sampling stage	Sample size
Bodnar et al. (27) (2007)	USA	Nested case–control	ELISA	Early & middle	274
Powe et al. (25) (2010)	USA	Nested case–control	LC–MS	Early	170
Baker et al. (39) (2010)	USA	Nested case–control	LC–MS	Middle	241
Wei et al. (28) (2012)	Canada	Cohort	CLIA	Early	729
Wei et al. (40) (2013)	Canada	Cohort	CLIA	Middle	729
Schneuer et al. (26) (2013)	Australia	Nested case–control	AIA	Early	3,937
Wetta et al. (24) (2014)	USA	Nested case–control	LC–MS	Middle	266
Achkar et al. (44) (2015)	Canada	Nested case–control	AIA	Early & middle	2,144
van Weert et al. (29) (2016)	Netherlands	Cohort	ELISA	Early	2074
Boyle et al. (45) (2016)	New Zealand	Cohort	LC–MS	Early & middle	1715
Baca et al. (41) (2010)	USA	Cohort	LC–MS	Middle	2,977
Zhao et al. (42) (2017)	China	Cohort	AIA	Middle	2,977
Magnus et al. (46) (2018)	Europe	MR	LC–MS	Early & middle	7,389
Benachi et al. (30) (2019)	Belgium/France	Nested case–control	RIA	Early	402
Álvarez-Fernández et al. (31) (2019)	Spain	Cross-sectional	ECLIA	Early	257
Flood-Nichols et al. (32) (2015)	USA	Cohort	ELISA	Early	310
Hemmingway et al. (47) (2018)	Ireland	Cohort	LC–MS	Early & middle	1754
Mirzakhani et al. (48) (2016)	USA	Nested case–control	CLIA	Early & middle	157
Scholl et al. (49) (2013)	USA	Cohort	LC–MS	Early & middle	1,141
Shand et al. (50) (2010)	Canada	Cohort	RIA	Early & middle	227
Gidlöf et al. (33) (2015)	Sweden	Nested case–control	CLIA	Early	157
Christoph et al. (51) (2020)	Switzerland	Cross-sectional	CLIA	Early & middle	1,153
Yue et al. (52) (2021)	China	Cohort	CLIA	Early & middle	7,976
Wang et al. (38) (2021)	China	Case–control	LC–MS/MS	Early	1,687
Vestergaard et al. (37) (2021)	Denmark	Cohort	HPLC-MS/MS	Early	225
Nema et al. (36) (2023)	India	Cohort	ELISA	Early	324
Ni et al. (35) (2021)	China	Cross-sectional	CLIA	Early	23,394
Malm et al. (34) (2023)	Sweden	Case–control	LC–MS/MS	Early	876
Zhou et al. (43) (2014)	China	Cohort	ECLIA	Middle	1,953

### Risk of bias of included studies

**Figure 2:**
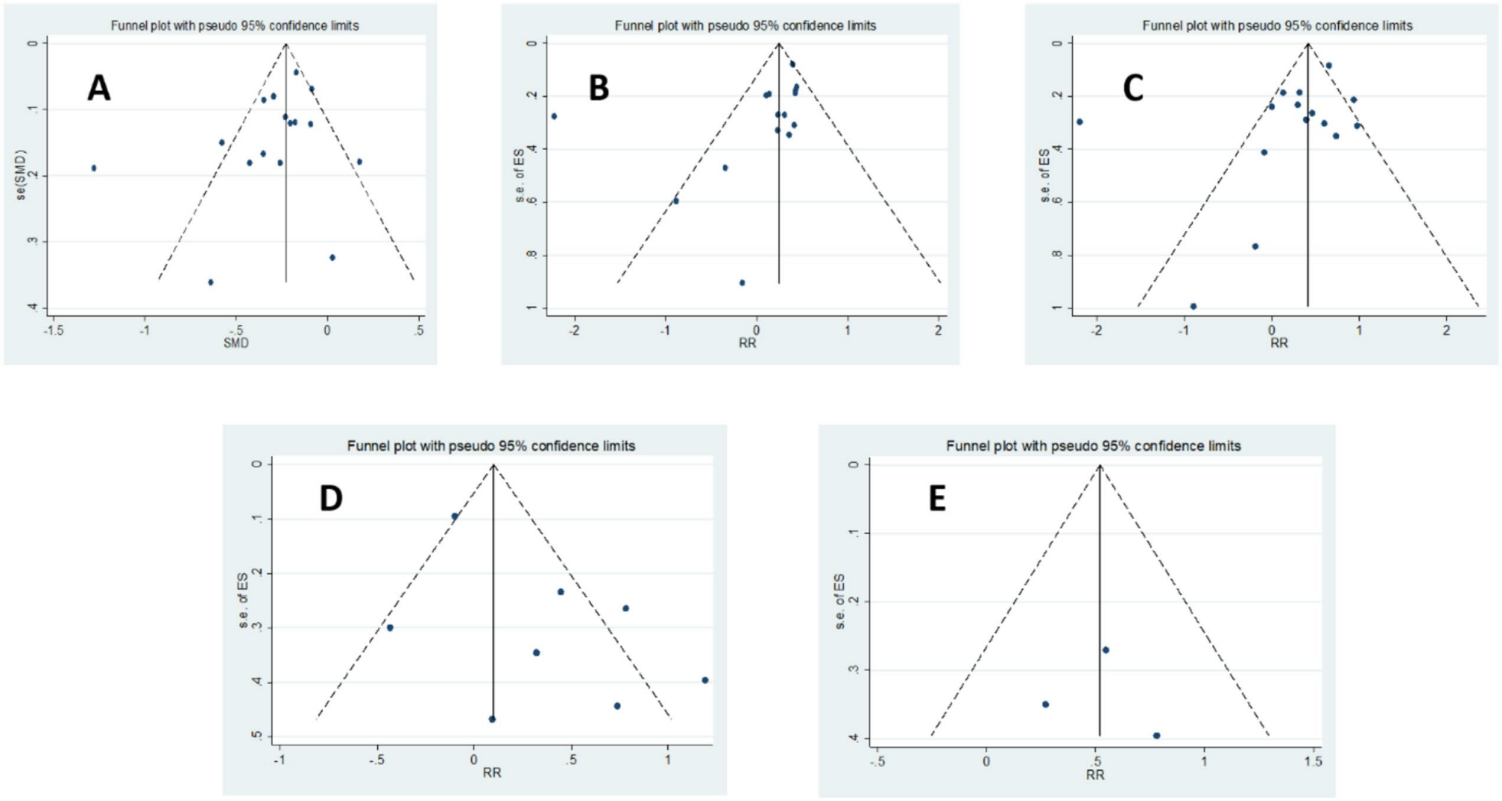
Funnel plot of the studies represented in the meta-analysis; **(A)** Vitamin D concentration in preeclampsia versus normal pregnancy, **(B)** Sufficient levels of Vitamin D (≥75 nmol/L) versus Insufficient levels of Vitamin D (50–75 nmol/L or 37.5–75 nmol/L), **(C)** Sufficient Levels of Vitamin D (≥75 nmol/L) versus Deficient Levels of Vitamin D (<50 nmol/L or <37.5 nmol/L), **(D)** Sufficient or Insufficient Levels of Vitamin D (≥50 nmol/L) versus Deficient Levels of Vitamin D (<50 nmol/L or < 37.5 nmol/L), **(E)** Sufficient Levels of Vitamin D (≥75 nmol/L) versus Insufficient or Deficient Levels of Vitamin D (<75 nmol/L).

The methodological quality of included studies was assessed using the JBI tool. All case–control studies achieved a high quality score of 9 out of 10. Cohort studies generally demonstrated excellent quality, with a score of 11 out of 11, except for Nema et al. (36) (score of 6/11) and Vestergaard et al. (37) (score of 9/11). Among the cross-sectional studies, Ni et al. (35) obtained a score of 8 out of 8, while the remaining two studies scored 6 out of 8. To assess publication bias, Egger’s regression, Begg’s test, and funnel plot analyses were conducted for all comparisons. Evidence of publication bias was detected in the comparison of sufficient versus insufficient vitamin D levels based on Begg’s test (*p* < 0.1) and in the comparison of sufficient versus insufficient or deficient levels of vitamin D based on Egger’s test and funnel plot asymmetry (Figure 2). These findings suggest that publication bias may have influenced the results. Furthermore, the trim-and-fill analysis for the mean difference outcome showed no imputed studies, indicating a low risk of publication bias; the pooled effect size remained stable after adjustment (mean difference: –4.009; 95% CI: −4.743 to −3.276). For the odds ratio outcomes, some imputed studies were identified. In the Sufficient vs. Insufficient category, two studies were imputed, and the pooled OR shifted slightly from 1.37 (95% CI: 1.13–1.61) to 1.33 (95% CI: 1.10–1.57). In the Sufficient vs. Deficient category, three studies were imputed, with the pooled OR changing from 1.43 (95% CI: 1.15–1.71) to 1.40 (95% CI: 1.13–1.68). In the Sufficient/Insufficient vs. Deficient category, three studies were imputed, and the pooled OR decreased marginally from 1.40 (95% CI: 1.16–1.65) to 1.38 (95% CI: 1.13–1.62). Finally, in the Sufficient vs. Insufficient/Deficient category, no studies were imputed, indicating stable results. Overall, these findings suggest that although minor adjustments occurred after imputing potentially missing studies, the effect sizes remained consistent, indicating that publication bias is unlikely to have influenced the conclusions of this meta-analysis in a meaningful way.

### Sensitivity studies

Sensitivity analysis indicated that the removal of any individual study or subgroup of studies had minimal impact on the pooled risk ratio and its confidence interval (Supplementary Figures S1, S2), suggesting the robustness of our findings.

### Principal findings

#### Vitamin D concentration in preeclampsia versus normal pregnancy

**Figure 3:**
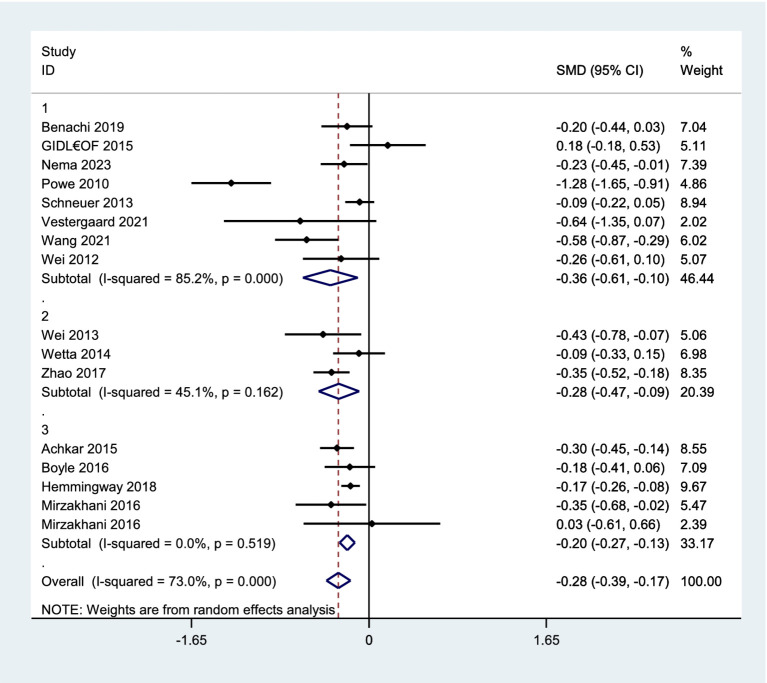
Forest plot for vitamin D concentration in preeclampsia versus normal pregnancy. 1. Early pregnancy, 2. Middle pregnancy, 3. Early or middle pregnancy.

A total of 20 studies reported the mean concentration of vitamin D among participants, out of which four (27, 31, 32, 41), did not provide the standard deviation of data. Finally, 16 studies (2, 24–26, 28, 30, 33, 36–38, 40, 42, 44, 45, 47, 48) comprising 24,247 pregnant women (including 1,133 with pre-eclampsia) were eligible for the meta-analysis comparing vitamin D status in pregnant women with and without pre-eclampsia. The analysis revealed that women with pre-eclampsia had significantly lower vitamin D levels compared to those without it (SMD, −0.28; 95% CI, −0.39, −0.17; *p*-value<0.001). A subgroup analysis showed that this observation persisted regardless of whether serum vitamin D was measured during the early and middle pregnancy (as shown in Figure 3). However, there was significant heterogeneity among these studies (*I*^2^ = 73.0%). Therefore, a random effects model was applied. Subgroup analyses confirmed this association across various methodological and clinical parameters, including assay type, vitamin D cut-off definitions, geographic region, and study design. Although effect sizes varied slightly among subgroups, the direction of association remained consistent. No heterogeneity was detected among subgroups (see Supplementary Table S2 for details).

#### Sufficient levels of vitamin D (≥75 nmol/L) versus insufficient levels of vitamin D (50–75 nmol/L or 37.5–75 nmol/L)

**Figure 4:**
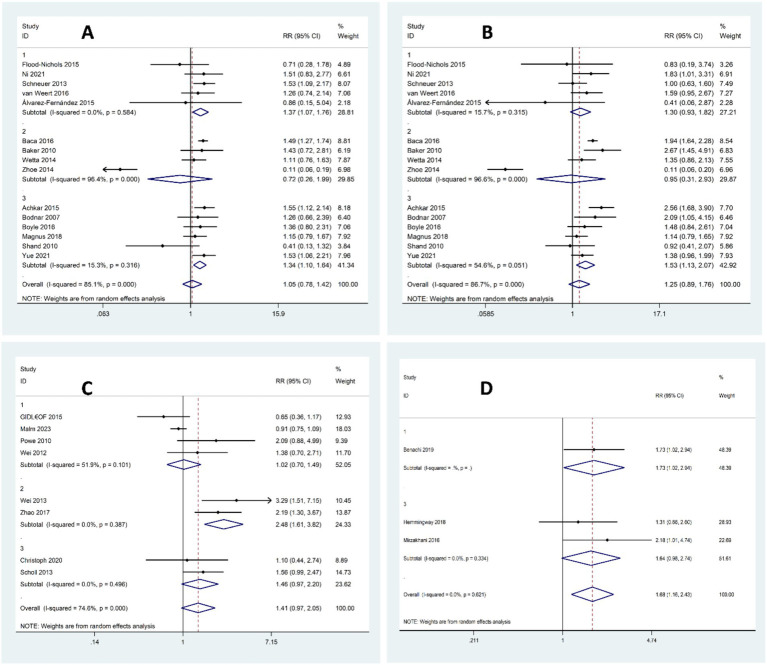
Forest plot for the risk of preeclampsia according to vitamin D status; **(A)** Sufficient Levels of Vitamin D (≥75 nmol/L) versus Insufficient levels of Vitamin D (50–75 nmol/L or 37.5–75 nmol/L), **(B)** Sufficient Levels of Vitamin D (≥75 nmol/L) versus Deficient Levels of Vitamin D (<50 nmol/L or <37.5 nmol/L), **(C)** Sufficient or Insufficient Levels of Vitamin D (≥50 nmol/L) versus Deficient Levels of Vitamin D (<50 nmol/L or < 37.5 nmol/L), **(D)** Sufficient Levels of Vitamin D (≥75 nmol/L) versus Insufficient or Deficient Levels of Vitamin D (<75 nmol/L).

A total of 27,448 pregnant women from 15 studies were included in this analysis, with 8,756 having sufficient levels of vitamin D and 18,692 having insufficient levels. There was a trend for an increased risk of preeclampsia in women with vitamin D insufficiency compared to women with sufficient levels, but it was not statistically significant (OR 1.05, 95% CI 0.78–1.42, *I*^2^ = 85.1%; *p*-value<0.001, random-effect). However, the subgroup analysis revealed a significant increase in the risk of preeclampsia in studies of women with insufficient vitamin D levels during early pregnancy or overlapping between early and middle pregnancy, but not in middle pregnancy alone (early pregnancy: OR 1.37, 95% CI 1.07–1.76, *I*^2^ = 0%; *p*-value = 0.584, random-effect; middle pregnancy: OR 0.72, 95% CI 0.26–1.99, *I*^2^ = 96.4%; *p*-value < 0.001, random-effect; early and middle pregnancy: OR 1.34, 95% CI 1.10–1.64, *I*^2^ = 15.3%; *p*-value = 0.316, random-effect), detailed in Figure 4A. Further subgroup analyses based on assay method, vitamin D cut-off definitions, region, and study design consistently supported the association between vitamin D insufficiency and increased risk of preeclampsia. No significant between-group heterogeneity was found (see Supplementary Table S3 for full subgroup results).

#### Sufficient levels of vitamin D (≥75 nmol/L) versus deficient levels of vitamin D (<50 nmol/L or <37.5 nmol/L)

In the next analysis that included a total of 33,321 pregnant women from 15 studies, 8,756 had sufficient levels of vitamin D while 24,565 had deficient levels. There was an overall trend indicating an increased risk of preeclampsia in women with vitamin D deficiency compared to those with sufficient levels, but the findings did not reach statistical significance *p*-value<0.001 (OR 1.25, 95% CI 0.89–1.76, *I*^2^ = 86.7%;, random-effect). The next subgroup analysis revealed a significant increase in the risk of preeclampsia in women with deficient Vitamin D levels during the overlapping period between early and middle pregnancy, but not in early or middle pregnancy alone (early pregnancy: OR 1.30, 95% CI 0.93–1.82, *I*^2^ = 15.7%; *p*-value = 0.315, random-effect; middle pregnancy: OR 0.95, 95% CI 0.31–2.93, *I*^2^ = 96.6%; *p*-value < 0.001, random-effect; early and middle pregnancy: OR 1.53, 95% CI 1.13–2.07, *I*^2^ = 54.6%; *p*-value = 0.061, random-effect), detailed in Figure 4B. Further subgroup analyses by assay method, cut-off definitions, region, and study design generally supported the association between vitamin D deficiency and elevated preeclampsia risk. However, no significant between-group heterogeneity was detected (see Supplementary Table S4 for detailed subgroup results).

#### Sufficient or insufficient levels of vitamin D (≥50 nmol/L) versus deficient levels of vitamin D (<50 nmol/L or < 37.5 nmol/L)

In the next analysis that included a total of 15,751 pregnant women from 8 studies, 4,952 had sufficient or insufficient levels of Vitamin D while 10,799 had deficient levels. While there was an overall trend for higher risk of preeclampsia in women with vitamin D deficiency compared to those with sufficient or insufficient levels, the results were not statistically significant (OR 1.41, 95% CI 0.97–2.05, *I*^2^ = 74.6%; *p*-value<0.001, random-effect). The next subgroup analysis showed a significant increase in preeclampsia risk only among women with deficient vitamin D levels in their middle pregnancy (early pregnancy: OR 1.02, 95% CI 0.70–1.49, *I*^2^ = 51.9%, *p*-value = 0.101, random-effect; middle pregnancy: OR 2.48, 95% CI 1.61–3.82, *I*^2^ = 0%; *p*-value = 0.387, random-effect; early and middle pregnancy: OR 1.41, 95% CI 0.97–2.05, *I*^2^ = 0%; *p*-value = 0.496, random-effect), detailed in Figure 4C. Further subgroup analyses by assay method, cut-off definitions, region, and study design generally supported the association between vitamin D deficiency and elevated preeclampsia risk. However, no significant between-group heterogeneity was detected (see Supplementary Table S5 for detailed subgroup results).

#### Sufficient levels of vitamin D (≥75 nmol/L) versus insufficient or deficient levels of vitamin D (<75 nmol/L)

In the next analysis that included a total of 2,313 pregnant women from three studies, 576 had sufficient levels of vitamin D and 1,737 had insufficient or deficient levels of vitamin D. The risk of preeclampsia was significantly higher in women with vitamin D status of insufficiency or deficiency compared to those with sufficient levels (OR 1.68, 95% CI 1.16–2.43, *I*^2^ = 0%; *p*-value = 0.621, fixed-effect), detailed in Figure 4D. Subgroup analyses revealed some variation based on assay method, threshold definitions, region, and study design. However, it should be noted that most subgroups included only one or two studies, which limits the robustness and generalizability of these subgroup findings (see Supplementary Table S6 for full subgroup results).

### Meta-regression analysis

Meta-regression analyses were conducted to examine whether assay method, deficiency cut-off, sufficiency cut-off, study region, or study design contributed to the observed heterogeneity (Supplementary Table S7). In most comparisons, none of these factors had a statistically significant effect on the results (all *p* > 0.05). An exception was found for the comparison between sufficient vitamin D levels (≥75 nmol/L) and insufficient or deficient levels (<75 nmol/L), where assay method (*p* = 0.037) and study region (*p* < 0.001) were significant predictors. These findings should be interpreted with caution, as most subgroups for these moderators included only one or two studies, which limits the strength and generalizability of the conclusions.

Moreover, an ordinal meta-regression across commonly applied thresholds (37.5, 50, and 75 nmol/L; REML) did not indicate a significant trend (*β* = 0.17, 95% CI − 0.27 to 0.61; Wald *χ*^2^ = 0.56; *p* = 0.455), with heterogeneity increasing slightly (τ^2^ from 0.0165 at baseline to 0.0214; residual *I*^2^ = 27.9%; adjusted *R*^2^ = 0%). In a sensitivity analysis restricted to uniform thresholds, studies using 50 nmol/L as the cut-off produced an overall OR of 1.23 (95% CI 0.51–1.95; τ^2^ = 0.3617; *I*^2^ = 61.4%; REML), while those applying 75 nmol/L yielded OR = 1.12 (95% CI 0.99–1.26; τ^2^ = 0.0120; *I*^2^ = 20.8%). These results were directionally consistent with the main model, suggesting that the findings are not dependent on the specific threshold applied.

The relationship between vitamin D thresholds and preeclampsia risk was further examined using bubble and Galbraith plots (Supplementary Figures S3, S4). In the bubble plot the linear prediction line was nearly flat, and confidence intervals remained wide, indicating no significant trend across increasing thresholds. In contrast, the Galbraith plot, suggested that studies with higher precision tended to report somewhat stronger associations. Although this pattern should be interpreted with caution given the variability among studies.

#### Comparison with existing literature

This meta-analysis of 29 observational studies investigated the association between vitamin D status during the early and middle pregnancy and the risk of preeclampsia using two approaches: (1) comparing mean vitamin D levels between pregnant women with and without preeclampsia, and (2) assessing the risk of preeclampsia based on predefined vitamin D deficiency, insufficiency, and normal ranges.

Our findings demonstrate significantly lower vitamin D levels in pregnant women who developed preeclampsia compared to those without. However, due to the observational nature of this study, a causal relationship between low vitamin D and preeclampsia cannot be definitively established. Nevertheless, our results align with previous meta-analyses linking vitamin D deficiency (below 50 nmol/L) to an increased risk of preeclampsia (15–17). These findings support the potential benefit of vitamin D supplementation in reducing this risk (53–55). One recent meta-analysis showed that when vitamin D levels falls below 40 nmol/L, the risk of preeclampsia dramatically increases (56). Consistent with previous research, a meta-analysis by (53) demonstrated a reduced risk of preeclampsia with vitamin D supplementation of up to 2,000 IU/day. His finding is supported by multiple meta-analyses of randomized controlled trials (RCTs) showing a similar association, despite variations in supplementation doses (54, 55, 57, 58). However, one meta-analysis of three RCTs reported no association between vitamin D supplementation and preeclampsia risk. The authors attributed this inconsistency to potential confounding effects of a single study and the limited sample size (59).

Establishing a clear cut-off point for vitamin D status is crucial for developing effective nutritional interventions to prevent preeclampsia. A significant challenge in this field has been the inconsistent use of vitamin D level thresholds across studies. Most studies included in this meta-analysis aligned with the Endocrine Society guidelines, categorizing vitamin D status as sufficient (>30 ng/mL or >75 nmol/L), insufficient (20–30 ng/mL or 50–75 nmol/L), or deficient (<20 ng/mL or <50 nmol/L) (23). Previous meta-analyses encompassing vitamin D measurements across the entire pregnancy period have consistently linked vitamin D levels below 50 nmol/L to an increased risk of preeclampsia (15–17). Narrowing down the time of measurement to the early and middle pregnancy allows for the use of preventive methods before the disease progresses. Interestingly, a meta-analysis conducted by Hu et al. (14), found that pregnant women with early or mid-pregnancy vitamin D levels below 50 nmol/L exhibited an increased risk of preeclampsia. Nevertheless, two studies reported no association between vitamin D levels and preeclampsia risk during pregnancy (60, 61). Our analysis revealed a potential trend suggesting that vitamin D levels exceeding 75 nmol/L during early or middle pregnancy might be associated with a reduced preeclampsia risk compared to lower levels. This association was most pronounced when comparing sufficient versus insufficient or deficient vitamin D status across three studies. While subgroup analysis by pregnancy stage strengthened these findings, the inconsistent reporting of pregnancy stages across studies limited definitive conclusions regarding stage-specific effects.

The discrepancies observed among meta-analyses can be attributed to several factors, primarily heterogeneity in study design and methodology. Variations in study type, timing of vitamin D measurement, assay methods, and definitions of vitamin D deficiency can significantly influence pooled results. Our subgroup analyses addressed some of these sources of heterogeneity, showing that the association between vitamin D status and preeclampsia was generally consistent despite variations in assay techniques (e.g., ELISA, LC–MS/MS), cut-off thresholds (e.g., 50 vs. 37.5 nmol/L), and study design (e.g., cohort, case–control). Results from the meta-regression further supported this consistency, as none of these factors showed statistically significant contributions to heterogeneity. Although adjusted odds ratios account for potential confounders, overadjustment may obscure true associations. Additionally, the meta-analysis methodology, including inclusion criteria and statistical models, can impact the overall findings.

The pathogenesis of preeclampsia is not yet fully understood. It is proposed that insufficient trophoblast cell invasion and defective remodeling of the uterine spiral arteries causes placental ischemia. This reduction in placental blood flow triggers an immune system imbalance, where there is an increase in proinflammatory CD4^+^ T cells and cytokines, and a decrease in regulatory T cells and anti-inflammatory cytokines. As a result, chronic inflammation develops, leading to oxidative stress, elevated proinflammatory cytokines, and the production of autoantibodies (62). Interestingly, low levels of vitamin D could further sensitize the endothelium to inflammation and induce oxidative stress (63). Conversely, adequate vitamin D has been found to affect the endovascular system directly by suppressing vascular wall cell proliferation and having immunomodulatory effects (64), and indirectly through the regulation of calcium levels and blood pressure (65). Therefore, inadequate amounts of vitamin D may contribute to the development of preeclampsia.

#### Strengths and limitations

Our meta-analysis has several strengths. By focusing on vitamin D measurements from early and middle pregnancy, we targeted a critical period for potential intervention. Additionally, incorporating both mean vitamin D levels and odds ratios enhanced the comprehensiveness of our analysis, given the diverse reporting of these data in the literature. Our use of three vitamin D status categories provided a more nuanced assessment compared to dichotomizing into deficient and non-deficient groups. Subgroup analyses and meta-regression across multiple parameters, including assay methods, vitamin D thresholds, region, and study design, further validated the consistency of findings.

This meta-analysis has also several limitations that should be addressed. Firstly, there was significant heterogeneity among studies for Preeclampsia risk regarding gestational weeks, study design, methods of vitamin D assessment, threshold for vitamin D status, and participants’ characteristics. Additionally, few studies specified or adjusted for the season of sampling, which is known to influence vitamin D concentrations. Secondly, to To minimize the influence of confounding factors, only adjusted RRs were entered into the analysis, but unidentified confounders could still skew the results. Some evidence of publication bias was detected in specific comparisons; however, trim-and-fill analysis showed few imputed studies and only minor shifts in pooled estimates. These small changes suggest that publication bias is unlikely to have significantly affected the overall conclusions. Thirdly, the ambiguous reporting of the pregnancy stage during which vitamin D was measured in some studies made it difficult to compare the association between vitamin D levels and preeclampsia during early versus middle pregnancy. Furthermore, none of the included studies distinguished between early-onset and late-onset preeclampsia, therefore we could not extract or analyze this data, which limited our ability to explore potential differences in the relationship between vitamin D deficiency and preeclampsia subtypes. Finally, although threshold modelling was attempted through ordinal and sensitivity approaches, continuous meta-regression remained limited by the small number of distinct and overlapping cut-offs, reducing statistical power.

#### Conclusion and implications

In summary, this systematic review and meta-analysis investigated the role of vitamin D status prior to the late pregnancy in the development of preeclampsia. Based on the evidence presented in this study, it is suggested that low concentrations of maternal vitamin D prior to the late stage of pregnancy could be associated with an increased risk of preeclampsia, but the significance of this association depends on the time of measurement including the season of the year. To fully understand the role of vitamin D in preventing and managing preeclampsia, there is a need for well-designed trials on vitamin D supplementation. These trials should focus on identifying the optimal time for vitamin D enrichment during pregnancy, as well as evaluating the cost-effectiveness of this approach. Future research should explore more specific areas, such as investigating vitamin D status at the preconception stage and its potential impact on the risk of preeclampsia. Additionally, studies should consider subtypes of preeclampsia (e.g., early- or late-onset) to better understand how vitamin D deficiency may influence the development of these subtypes. Multi-center, multinational studies are recommended to further examine the impact of geographic, ethnic, and lifestyle factors.

## Data Availability

The original contributions presented in the study are included in the article/Supplementary material, further inquiries can be directed to the corresponding author.

## Footnotes

^1^ https://osf.io/qwh6a

^2^ https://jbi.global/critical-appraisal-tools
