# Supplementary material for: Vitamin D levels in early and middle pregnancy and preeclampsia: a systematic review and meta-analysis
Source: Front Med (Lausanne). 2025 Oct 23;12:1535359. doi: 10.3389/fmed.2025.1535359 (PMC12588914; doi:10.3389/fmed.2025.1535359)
Supplement: Supplementary file 1 [file Data_Sheet_1.zip › Data Sheet 1.ZIP!/Supplementary Material/PRISMA Checklist-vitdpreec.pdf]

PRISMA 2020 Main Checklist

Topic	No.	Item	Location where item is reported
**TITLE**			
**Title**	1	Identify the report as a systematic review.	Title page
**ABSTRACT**			
**Abstract**	2	See the PRISMA 2020 for Abstracts checklist	
**INTRODUCTION**			
**Rationale**	3	Describe the rationale for the review in the context of existing knowledge.	Introduction, paragraphs 1–2
**Objectives**	4	Provide an explicit statement of the objective(s) or question(s) the review addresses.	Introduction, final paragraph
**METHODS**			
**Eligibility criteria**	5	Specify the inclusion and exclusion criteria for the review and how studies were grouped for the syntheses.	Methods, "Eligibility criteria"
**Information sources**	6	Specify all databases, registers, websites, organisations, reference lists and other sources searched or consulted to identify studies. Specify the date when each source was last searched or consulted.	Methods, "Eligibility criteria, information sources, search strategy"
**Search strategy**	7	Present the full search strategies for all databases, registers and websites, including any filters and limits used.	Table 1
**Selection process**	8	Specify the methods used to decide whether a study met the inclusion criteria of the review, including how many reviewers screened each record and each report retrieved, whether they worked independently, and if applicable, details of automation tools used in the process.	Methods, "Study selection, Data extraction and Study quality assessment"
**Data collection process**	9	Specify the methods used to collect data from reports, including how many reviewers collected data from each report, whether they worked independently, any processes for obtaining or confirming data from study investigators, and if applicable, details of automation tools used in the process.	Methods, "Study selection, Data extraction and Study quality assessment"
**Data items**	10a	List and define all outcomes for which data were sought. Specify whether all results that were compatible with each outcome domain in each study were sought (e.g. for all measures, time points, analyses), and if not, the methods used to decide which results to collect.	Methods, "Study selection, Data extraction and Study quality assessment"
	10b	List and define all other variables for which data were sought (e.g. participant and intervention characteristics, funding sources). Describe any assumptions made about any missing or unclear information.	Methods, "Study selection, Data extraction and Study quality assessment"

Topic	No.	Item	Location where item is reported
**Study risk of bias assessment**	11	Specify the methods used to assess risk of bias in the included studies, including details of the tool(s) used, how many reviewers assessed each study and whether they worked independently, and if applicable, details of automation tools used in the process.	Methods, "Study selection, Data extraction and Study quality assessment"
**Effect measures**	12	Specify for each outcome the effect measure(s) (e.g. risk ratio, mean difference) used in the synthesis or presentation of results.	Methods, "Data synthesis" (first paragraph)
**Synthesis methods**	13a	Describe the processes used to decide which studies were eligible for each synthesis (e.g. tabulating the study intervention characteristics and comparing against the planned groups for each synthesis (item 5)).	Methods, "Data synthesis"
	13b	Describe any methods required to prepare the data for presentation or synthesis, such as handling of missing summary statistics, or data conversions.	Methods, "Data synthesis"
	13c	Describe any methods used to tabulate or visually display results of individual studies and syntheses.	Methods, "Data synthesis" (forest plots mentioned)
	13d	Describe any methods used to synthesize results and provide a rationale for the choice(s). If meta-analysis was performed, describe the model(s), method(s) to identify the presence and extent of statistical heterogeneity, and software package(s) used.	Methods, "Data synthesis" (random-effects model, I^2, STATA 13.1)
	13e	Describe any methods used to explore possible causes of heterogeneity among study results (e.g. subgroup analysis, meta-regression).	Methods, "Data synthesis"
	13f	Describe any sensitivity analyses conducted to assess robustness of the synthesized results.	Methods, "Data synthesis"
**Reporting bias assessment**	14	Describe any methods used to assess risk of bias due to missing results in a synthesis (arising from reporting biases).	Methods, "Data synthesis"
**Certainty assessment**	15	Describe any methods used to assess certainty (or confidence) in the body of evidence for an outcome.	Methods, "Data synthesis" (final paragraph); Introduction (last sentence)
**RESULTS**			
**Study selection**	16a	Describe the results of the search and selection process, from the number of records identified in the search to the number of studies included in the review, ideally using a flow diagram.	Results, "Study selection"; Figure 1
	16b	Cite studies that might appear to meet the inclusion criteria, but which were excluded, and explain why they were excluded.	Figure 1 (flow diagram shows exclusions)
**Study characteristics**	17	Cite each included study and present its characteristics.	Results, "Study characteristics"; Table 2
**Risk of bias in studies**	18	Present assessments of risk of bias for each included study.	Results, "Risk of bias of included studies"; Supplementary file

Topic	No.	Item	Location where item is reported
**Results of individual studies**	19	For all outcomes, present, for each study: (a) summary statistics for each group (where appropriate) and (b) an effect estimate and its precision (e.g. confidence/credible interval), ideally using structured tables or plots.	Forest Plots (Figs 2–3); Supplementary material
**Results of syntheses**	20a	For each synthesis, briefly summarise the characteristics and risk of bias among contributing studies.	Results, “Risk of bias of included studies”
	20b	Present results of all statistical syntheses conducted. If meta-analysis was done, present for each the summary estimate and its precision (e.g. confidence/credible interval) and measures of statistical heterogeneity. If comparing groups, describe the direction of the effect.	Results, in all four major comparison subsections; Figs 2–3
	20c	Present results of all investigations of possible causes of heterogeneity among study results.	Results, all subgroup analyses (e.g., by pregnancy stage, region, cut-off), Supplementary material
	20d	Present results of all sensitivity analyses conducted to assess the robustness of the synthesized results.	Results, “Sensitivity studies”, Supplementary material
**Reporting biases**	21	Present assessments of risk of bias due to missing results (arising from reporting biases) for each synthesis assessed.	Results, “Risk of bias of included studies” (Egger’s, Begg’s, funnel plot)
**Certainty of evidence**	22	Present assessments of certainty (or confidence) in the body of evidence for each outcome assessed.	Results, “Risk of bias of included studies”
**DISCUSSION**			
**Discussion**	23a	Provide a general interpretation of the results in the context of other evidence.	Discussion, first half
	23b	Discuss any limitations of the evidence included in the review.	Discussion, limitations section
	23c	Discuss any limitations of the review processes used.	Discussion, limitations section
	23d	Discuss implications of the results for practice, policy, and future research.	Conclusion and Implications
**OTHER INFORMATION**			
**Registration and protocol**	24a	Provide registration information for the review, including register name and registration number, or state that the review was not registered.	Methods, first paragraph (“registered on the Open Science Framework”)
	24b	Indicate where the review protocol can be accessed, or state that a protocol was not prepared.	Methods, first paragraph
	24c	Describe and explain any amendments to information provided at registration or in the protocol.	Not applicable

Topic	No.	Item	Location where item is reported
**Support**	25	Describe sources of financial or non-financial support for the review, and the role of the funders or sponsors in the review.	Funding: “None”
**Competing interests**	26	Declare any competing interests of review authors.	Competing interests: “None”
**Availability of data, code and other materials**	27	Report which of the following are publicly available and where they can be found: template data collection forms; data extracted from included studies; data used for all analyses; analytic code; any other materials used in the review.	Data Availability Statement: “Available upon request”

From: Page MJ, McKenzie JE, Bossuyt PM, Boutron I, Hoffmann TC, Mulrow CD, et al. The PRISMA 2020 statement: an updated guideline for reporting systematic reviews. MetaArXiv. 2020, September 14. DOI: 10.31222/osf.io/v7gm2. For more information, visit: www.prisma-statement.org
