# Supplementary material for: Vitamin D levels in early and middle pregnancy and preeclampsia: a systematic review and meta-analysis
Source: Front Med (Lausanne). 2025 Oct 23;12:1535359. doi: 10.3389/fmed.2025.1535359 (PMC12588914; doi:10.3389/fmed.2025.1535359)
Supplement: Supplementary file 1 [file Data_Sheet_1.zip › Data Sheet 1.ZIP!/Supplementary Material/Tables and Figures.docx]

**Table S1. Detailed Characteristics of Included Studies on Maternal Vitamin D Levels and Preeclampsia Risk**

| Author, Year | Country, Study Type | Method of Measurement | Stage of Sampling | Sample Size | Categories of Vitamin D Level (nmol/L) | Adjusted Variables |
| --- | --- | --- | --- | --- | --- | --- |
| Bodnar et al. (2007) | USA, Nested case-control | ELISA | Early and middle pregnancy | 274 | >75, 37.5–75, <37.5 | Race/ethnicity, season, sample, gestational age, pre-pregnancy BMI, education |
| Powe et al. (2010) | USA, Nested case-control | LC-MS | Early pregnancy | 170 | >37.5, <37.5 | BMI, nonwhite race, summer blood collection |
| Baker et al. (2010) | USA, Nested case-control | LC-MS | Middle pregnancy | 241 | ≥75, 50–75, <50 | Season, maternal age, multiparity, BMI, gestational age |
| Wei et al. (2012) | Canada, Cohort | CLIA | Early pregnancy | 729 | ≥50, <50 | Maternal age, smoking, BMI, season |
| Wei et al. (2013) | Canada, Cohort | CLIA | Middle pregnancy | 729 | ≥50, <50 | Maternal age, smoking, BMI, season |
| Schneuer et al. (2013) | Australia, Nested case-control | AIA | Early pregnancy | 3937 | >75, 37.5–75, <37.5 | Maternal age, parity, smoking, weight, HTN, diabetes, season, country of birth, SES |
| Wetta et al. (2014) | USA, Nested case-control | LC-MS | Middle pregnancy | 266 | ≥75, 37.5–75, <37.5 | Age, race/ethnicity, parity, preterm birth history, weight, smoking, GA, season |
| Achkar et al. (2015) | Canada, Nested case-control | AIA | Early and middle pregnancy | 2144 | ≥50, 30–50, ≤30 | BMI, parity, age, smoking, season, year, GA, study site |
| van Weert et al. (2016) | Netherlands, Cohort | ELISA | Early pregnancy | 2074 | >50, 30–50, <30 | Age, ethnicity, BMI, smoking, education |
| Boyle et al. (2016) | New Zealand, Cohort | LC-MS | Early and middle pregnancy | 1715 | >75, 50–74.9, 25–49.9, <25 | BMI, ethnicity |
| Baca et al. (2010) | USA, Cohort | LC-MS | Middle pregnancy | 2977 | >75, 50–75, <50 | Year, lab batch, GA, season, race/ethnicity, age, smoking, BMI, insurance, education, marital status, parity |
| Zhao et al. (2017) | China, Cohort | AIA | Middle pregnancy | 2977 | ≥75, 50–75, <50 | BMI, maternal age, parity, season |
| Magnus et al. (2018) | Europe, MR | LC-MS | Early and middle pregnancy | 7389 | >75, 50–75, <50 | Age, parity, BMI, education, smoking, calcium, GA |
| Benachi et al. (2019) | Belgium/France, Nested case-control | RIA | Early pregnancy | 402 | <75, ≥75 | Parity, age, BMI, season, skin color |
| Álvarez-Fernández (2019) | Spain, Cross-sectional | ECLIA | Early pregnancy | 257 | ≥75, 52.5–72.5, ≤50 | None |
| Flood-Nichols et al. (2015) | USA, Cohort | ELISA | Early pregnancy | 310 | >75, 52.5–72.5, <50 | BMI, season, ethnicity, tobacco use |
| Hemmingway et al. (2018) | Ireland, Cohort | LC-MS | Early and middle pregnancy | 1754 | ≥75, <75 | BMI, age, education, supplementation |
| Mirzakhani et al. (2016) | USA, Nested case-control | CLIA | Early and middle pregnancy | 157 | <75, ≥75 | Gravidity, age, race, BMI, study group, education, income, center |
| Scholl et al. (2013) | USA, Cohort | LC-MS | Early and middle pregnancy | 1141 | ≥50, <50 | Age, parity, ethnicity, BMI, smoking, GA |
| Shand et al. (2010) | Canada, Cohort | RIA | Early and middle pregnancy | 227 | >75, 50–75, <50 | Age, ethnicity, parity, BMI, season, multivitamin use, smoking |
| Gidlöf et al. (2015) | Sweden, Nested case-control | CLIA | Early pregnancy | 157 | ≥50, <50 | None |
| Christoph et al. (2020) | Switzerland, Cross-sectional | CLIA | Early and middle pregnancy | 1153 | ≥50, <50 | None |
| Yue et al. (2021) | China, Cohort | CLIA | Early and middle pregnancy | 7976 | <25, 25–50, 50–75, ≥75 | Age, parity, BMI, BP, cholesterol, triglycerides, Hcy, folate |
| Wang et al. (2021) | China, Case-control | LC-MS/MS | Early pregnancy | 1687 | Not specified | None |
| Vestergaard et al. (2021) | Denmark, Cohort | HPLC-MS/MS | Early pregnancy | 225 | ≥75, <75 | None |
| Nema et al. (2023) | India, Cohort | ELISA | Early pregnancy | 324 | Not reported | Not reported |
| Ni et al. (2021) | China, Cross-sectional | CLIA | Early pregnancy | 23,394 | >75, 50–75, <50 | Age, BMI, fetus sex, season, parity |
| Malm et al. (2023) | Sweden, Case-control | LC-MS/MS | Early pregnancy | 876 | <75 grouped as: 30.1, 30.1–49.0, 49.0–66.9, 66.9 | Maternal BMI, smoking, parity |
| Zhou et al. (2014) | China, Cohort | ECLIA | Middle pregnancy | 1953 | ≥75, 52.5–72.5, <50 | Age, BP, BMI, calcium |

**Table S2.** Subgroup analysis of vitamin D concentration in preeclampsia versus normal pregnancy.

| Study group | Number of studies | MD (95% CI) | P effect | I² (%) | P between group |
| --- | --- | --- | --- | --- | --- |
| Assay Method | 14 | -4.30 [-5.31, -3.29] | p < 0.001 | 13.77 | 0.69 |
| AIA (Automated Immunoassay) | 3 | -4.46 [-6.06, -2.87] | p < 0.001 | 0 |  |
| CLIA (Chemiluminescent Immunoassay) | 5 | -3.99 [-9.29, 1.31] | p = 0.14 | 52.03 |  |
| ELISA (Enzyme-Linked Immunosorbent Assay) | 1 | -10.48 [-20.42, -0.54] | p = 0.04 | 0 |  |
| LC-MS / LC-MS/MS / HPLC-MS/MS | 5 | -4.30 [-6.48, -2.12] | p < 0.001 | 58.21 |  |
| Deficiency Cut-off | 8 | 3.73 [ -4.54, -2.94] | p < 0.001 | 0 | 0.85 |
| Deficiency <37.5 nmol/L | 4 | -3.59 [-4.46, -2.72] | p < 0.001 | 0 |  |
| Deficiency <50 nmol/L | 4 | -3.98 [-7.85, -0.11] | p = 0.04 | 51.41 |  |
| Sufficiency Cut-off | 15 | -4.78 [-5.89, -3.66] | p < 0.001 | 0 | 0.70 |
| Sufficiency ≥50 nmol/L | 4 | -4.02 [-8.03, -0.02] | p = 0.05 | 52.4 |  |
| Sufficiency ≥75 nmol/L | 11 | -4.84 [-6.13, -3.56] | p < 0.001 | 0 |  |
| Region | 11 | -3.84 [-4.68, -3.01] | p < 0.001 | 0.82 | 0.81 |
| Europe | 4 | -3.55 [-7.86, 0.76] | p = 0.11 | 49.04 |  |
| North America | 7 | -4.11 [-5.47, -2.76] | p < 0.001 | 10.61 |  |
| Study Design | 15 | -3.95 [-4.73, -3.18] | p < 0.001 | 1.58 | 0.10 |
| Case-Control | 8 | -4.98 [-6.43, -3.53] | p < 0.001 | 0 |  |
| Cohort | 7 | -3.55 [-4.40, -2.70] | p < 0.001 | 0 |  |

**Table S3.** Sufficient vitamin D levels (≥75 nmol/L) versus insufficient levels (50–75 nmol/L or 37.5–75 nmol/L).

| Study group | Number of studies | MD (95% CI) | P effect | I² (%) | P between group |
| --- | --- | --- | --- | --- | --- |
| Assay Method | 8 | 1.39 [1.14, 1.64] | p = 0 | 0% | p = 0.69 |
| AIA | 2 | 2.06 [0.54, 3.59] | p = 0.01 | 60.85% |  |
| CLIA | 1 | 1.55 [0.92, 2.18] | p = 0 | 0% |  |
| ELISA | 1 | 1.36 [-0.34, 3.06] | p = 0.12 | 0% |  |
| LC-MS / LC-MS/MS / HPLC-MS/MS | 4 | 1.27 [0.96, 1.59] | p = 0 | 0% |  |
| Deficiency Cut-off | 8 | 1.40 [1.14, 1.66] | <0.001 | 0% | 0.65 |
| Deficiency <37.5 nmol/L | 2 | 1.34 [0.90, 1.79] | <0.001 | 0% |  |
| Deficiency <50 nmol/L | 4 | 1.38 [1.05, 1.70] | <0.001 | 0% |  |
| Other thresholds | 2 | 2.20 [0.44, 3.96] | 0.01 | 46.5% |  |
| Sufficiency Cut-off | 10 | 1.36 [1.14, 1.58] | <0.001 | 0 | 0.58 |
| Sufficiency ≥50 nmol/L | 1 | 1.51 [0.93, 2.09] | <0.001 | 0 |  |
| Sufficiency ≥75 nmol/L | 9 | 1.33 [1.09, 1.58] | <0.001 | 0 |  |
| Region | 9 | 1.39 [1.15, 1.63] | <0.001 | 0% | 0.63 |
| East Asia | 3 | 1.61 [1.11, 2.11] | <0.001 | 0% |  |
| Europe | 1 | 1.29 [0.70, 1.88] | <0.001 | 0% |  |
| North America | 5 | 1.34 [1.03, 1.65] | <0.001 | 0% |  |
| Study Design | 9 | 1.37 [1.13, 1.61] | <0.001 | 0% | 0.99 |
| Case-Control | 3 | 1.37 [0.94, 1.81] | <0.001 | 0% |  |
| Cohort | 6 | 1.37 [1.08, 1.66] | <0.001 | 0% |  |

**Table S4.** Sufficient vitamin D levels (≥75 nmol/L) versus deficient levels (<50 nmol/L or <37.5 nmol/L).

| Study group | Number of studies | MD (95% CI) | P effect | I² (%) | P between group |
| --- | --- | --- | --- | --- | --- |
| Assay Method | 13 | 1.37 [1.11, 1.64] | 0 | 0 | 0.15 |
| AIA | 2 | 2.51 [1.45, 3.57] | 0 | 0 |  |
| CLIA | 3 | 1.48 [0.95, 2.01] | 0 | 0 |  |
| ELISA | 3 | 1.26 [0.60, 1.91] | 0 | 0 |  |
| LC-MS / LC-MS/MS / HPLC-MS/MS | 5 | 1.22 [0.85, 1.59] | 0 | 0 |  |
| Deficiency Cut-off | 13 | 1.49 [1.12, 1.85] | 0 | 8.74 | 0.67 |
| Deficiency defined as <37.5 nmol/L | 4 | 1.53 [0.71, 2.34] | 0 | 24.11 |  |
| Deficiency defined as <50 nmol/L | 7 | 1.39 [0.97, 1.81] | 0 | 1.66 |  |
| Other thresholds | 2 | 2.20 [0.44, 3.96] | 0.01 | 46.54 |  |
| Sufficiency Cut-off | 15 | 1.35 [1.12, 1.59] | 0 | 0 | 0.72 |
| Sufficiency defined as ≥50 nmol/L | 4 | 1.44 [0.91, 1.97] | 0 | 0 |  |
| Sufficiency defined as ≥75 nmol/L | 11 | 1.33 [1.07, 1.59] | 0 | 0 |  |
| Region | 15 | 1.39 [1.13, 1.64] | 0 | 0 | 0.44 |
| East Asia | 3 | 1.64 [1.13, 2.16] | 0 | 0 |  |
| Europe | 3 | 1.23 [0.84, 1.61] | 0 | 0 |  |
| Study Design | 14 | 1.43 [1.16, 1.71] | 0 | 0 | 0.70 |
| Case-Control | 5 | 1.62 [0.76, 2.47] | 0 | 23.59 |  |
| Cohort | 9 | 1.44 [1.12, 1.75] | 0 | 0 |  |

**Table S5.** Sufficient or insufficient vitamin D levels (≥50 nmol/L) versus deficient levels (<50 nmol/L or <37.5 nmol/L).

| Study group | Number of studies | MD (95% CI) | P effect | I² (%) | P between group |
| --- | --- | --- | --- | --- | --- |
| Assay Method | 10 | 1.36 [1.12, 1.60] | 0 | 0 | 0.56 |
| AIA | 3 | 1.93 [0.92, 2.94] | 0 | 56.25 |  |
| CLIA | 3 | 1.48 [0.95, 2.01] | 0 | 0 |  |
| ELISA | 3 | 1.26 [0.60, 1.91] | 0 | 0 |  |
| LC-MS / LC-MS/MS / HPLC-MS/MS | 5 | 1.22 [0.85, 1.59] | 0 | 0 |  |
| Deficiency Cut-off | 13 | 1.40 [1.12, 1.68] | 0 | 0 | 0.65 |
| Deficiency <37.5 nmol/L | 5 | 1.34 [0.95, 1.74] | 0 | 0 |  |
| Deficiency <50 nmol/L | 7 | 1.39 [0.97, 1.81] | 0 | 1.66 |  |
| Other thresholds | 2 | 2.20 [0.44, 3.96] | 0.01 | 46.54 |  |
| Sufficiency Cut-off | 16 | 1.34 [1.13, 1.56] | 0 | 0 | 0.70 |
| Sufficiency ≥50 nmol/L | 4 | 1.44 [0.91, 1.97] | 0 | 0 |  |
| Sufficiency ≥75 nmol/L | 12 | 1.32 [1.09, 1.56] | 0 | 0 |  |
| Region | 15 | 1.39 [1.13, 1.64] | 0 | 0 | 0.44 |
| East Asia | 3 | 1.64 [1.13, 2.16] | 0 | 0 |  |
| Europe | 3 | 1.23 [0.84, 1.61] | 0 | 0 |  |
| North America | 9 | 1.42 [0.97, 1.88] | 0 | 0 |  |
| Study Design | 15 | 1.41 [1.16, 1.65] | 0 | 0 | 0.77 |
| Case-Control | 6 | 1.36 [0.97, 1.75] | 0 | 0 |  |
| Cohort | 9 | 1.44 [1.12, 1.75] | 0 | 0 |  |

**Table S6.** Sufficient vitamin D levels (≥75 nmol/L) versus insufficient or deficient levels (<75 nmol/L).

| Study group | Number of studies | MD (95% CI) | P effect | I² (%) | P between group |
| --- | --- | --- | --- | --- | --- |
| Assay Method | 3 | 0.54 [0.14, 0.94] | 0.01 | 54.68 | 0.04 |
| AIA | 1 | 0.90 [0.48, 1.32] | 0.00003 | 0 | — |
| CLIA | 2 | 0.33 [-0.01, 0.66] | 0.06 | 0 | — |
| Deficiency Cut-off | 3 | 0.86 [0.56, 1.16] | <0.001 | 58.78 | 0.09 |
| Deficiency <37.5 nmol/L | 1 | 0.90 [0.48, 1.32] | <0.001 | 0 | — |
| Deficiency <50 nmol/L | 1 | 0.40 [-0.15, 0.95] | 0.15 | 0 | — |
| Other thresholds | 1 | 1.00 [0.96, 1.04] | <0.001 | 0 | — |
| Sufficiency Cut-off | 4 | 0.55 [0.29, 0.82] | <0.001 | 33.65 | 0.57 |
| Sufficiency ≥50 nmol/L | 1 | 0.40 [-0.15, 0.95] | 0.15 | 0 | — |
| Sufficiency ≥75 nmol/L | 3 | 0.58 [0.25, 0.92] | <0.001 | 51.36 | — |
| Region | 4 | 0.62 [0.26, 0.97] | <0.001 | 80.21 | <0.001 |
| East Asia | 1 | 1.00 [0.96, 1.04] | <0.001 | 0 | — |
| Europe | 2 | 0.52 [0.22, 0.82] | <0.001 | 0 | — |
| North America | 1 | 0.28 [-0.15, 0.71] | 0.20 | 0 | — |
| Study Design | 4 | 0.72 [0.40, 1.05] | <0.001 | 79.39 | 0.01 |
| Case-Control | 3 | 0.58 [0.25, 0.92] | <0.001 | 51.36 | — |
| Cohort | 1 | 1.00 [0.96, 1.04] | <0.001 | 0 | — |

**Table S7. Meta-regression analyses evaluating heterogeneity in the association between vitamin D status and preeclampsia.**

| 1. Vitamin D concentration in preeclampsia vs. normal pregnancy | Comparison | τ² before | τ² after | Adjusted R² | Residual I² | p-value |
| --- | --- | --- | --- | --- | --- | --- |
|  | Assay | 0.016 | 1.746 | 0.00% | 31.27% | 0.769 |
|  | Region | 0.016 | 0.638 | 0.00% | 11.16% | 0.830 |
|  | Deficiency cut-off | 0.016 | 1.1e-07 | 0.00% | 0.00% | 0.410 |
|  | Sufficiency cut-off | 0.016 | 4.0e-08 | 0.00% | 0.00% | 0.834 |
|  | Study Design | 0.016 | 1.3e-07 | 0.00% | 0.00% | 0.096 |
| 2. Sufficient (≥75 nmol/L) vs. Insufficient (50–75 nmol/L or 37.5–75 nmol/L) | Assay | 0.016 | 6.0e-08 | 0.00% | 0.00% | 0.210 |
|  | Deficiency cut-off | 0.016 | 8.9e-07 | 0.00% | 0.00% | 0.497 |
|  | Region | 0.016 | 1.2e-07 | 0.00% | 0.00% | 0.579 |
|  | Study design | 0.016 | 1.4e-08 | 0.00% | 0.00% | 0.427 |
|  | Sufficiency cut-off | 0.016 | 3.6e-08 | 0.00% | 0.00% | 0.987 |
| 3. Sufficient (≥75 nmol/L) vs. Deficient (<50 nmol/L or <37.5 nmol/L) | Assay | 0.016 | 1.7e-07 | 0.00% | 0.00% | 0.066 |
|  | Deficiency cut-off | 0.016 | 0.083 | 0.00% | 15.73% | 0.538 |
|  | Region | 0.016 | 9.0e-08 | 0.00% | 0.00% | 0.717 |
|  | Study design | 0.016 | 2.3e-07 | 0.00% | 0.00% | 0.596 |
|  | Sufficiency cut-off | 0.016 | 6.2e-07 | 0.00% | 0.00% | 0.985 |
| 4. Sufficient/Insufficient (≥50 nmol/L) vs. Deficient (<50 nmol/L or <37.5 nmol/L) | Assay | 0.016 | 3.1e-09 | 0.00% | 0.00% | 0.243 |
|  | Deficiency cut-off | 0.016 | 0.0003 | 0.00% | 0.09% | 0.452 |
|  | Region | 0.016 | 9.0e-08 | 0.00% | 0.00% | 0.696 |
|  | Study design | 0.016 | 4.8e-08 | 0.00% | 0.00% | 0.596 |
|  | Sufficiency cut-off | 0.016 | 6.1e-07 | 0.00% | 0.00% | 0.766 |
| 5. Sufficient (≥75 nmol/L) vs. Insufficient/Deficient (<75 nmol/L) | Assay | 0.016 | 1.8e-07 | 0.00% | 0.00% | 0.037 |
|  | Deficiency cut-off | 0.016 | 0.141 | 0.00% | 70.14% | 0.805 |
|  | Region | 0.016 | 2.8e-07 | 0.00% | 0.00% | 0.454 |
|  | Study design | 0.016 | 0.044 | 0.00% | 51.36% | <0.001 |
|  | Sufficiency cut-off | 0.016 | 0.080 | 0.00% | 79.39% | 0.125 |

**Figure S1.** Sensitivity analysis of vitamin D concentration in preeclampsia versus normal pregnancy.

**Figure S2.** Sensitivity analysis of preeclampsia risk according to vitamin D status:
A) Sufficient (≥75 nmol/L) vs. insufficient (50–75 nmol/L or 37.5–75 nmol/L)
B) Sufficient (≥75 nmol/L) vs. deficient (<50 nmol/L or <37.5 nmol/L)
C) Sufficient/insufficient (≥50 nmol/L) vs. deficient (<50 nmol/L or <37.5 nmol/L)
D) Sufficient (≥75 nmol/L) vs. insufficient/deficient (<75 nmol/L)

**Figure S3. Bubble Plot of Odds Ratio Across Vitamin D Cut-off Values:** Blue bubbles show individual studies, with size according to inverse variance weight. The gray shaded area indicates the 95% confidence interval. The almost flat red line shows the fitted linear prediction, which does not suggest a significant trend across the thresholds.

**Figure S4. Galbraith Plot of Standardized Effect Size vs. Precision:** The X-axis shows study precision, and the Y-axis shows standardized effect size. Blue bubbles indicate individual studies, positioned by precision and effect size. The gray shaded area marks the 95% confidence interval around the regression. The red line represents the fitted regression, suggesting a positive trend. The black horizontal line at y = 0 shows the line of no effect.
